# High Prevalence of Cardiometabolic Comorbidities Among Children and Adolescents With Severe Obesity From a Large Metropolitan Centre (Hangzhou, China)

**DOI:** 10.3389/fendo.2022.807380

**Published:** 2022-05-19

**Authors:** Jinling Wang, Hu Lin, Valentina Chiavaroli, Binghan Jin, Jinna Yuan, Ke Huang, Wei Wu, Guanping Dong, José G. B. Derraik, Junfen Fu

**Affiliations:** ^1^ Department of Endocrinology, The Children’s Hospital, Zhejiang University School of Medicine, National Clinical Research Center for Child Health, Hangzhou, China; ^2^ Neonatal Intensive Care Unit, Pescara Public Hospital, Pescara, Italy; ^3^ Liggins Institute, University of Auckland, Auckland, New Zealand; ^4^ Department of Women’s and Children’s Health, Uppsala University, Uppsala, Sweden; ^5^ Environmental and Occupational Health Sciences and Non-Communicable Diseases Research Group, Research Institute for Health Sciences, Chiang Mai University, Chiang Mai, Thailand

**Keywords:** abnormal liver function, acanthosis nigricans, blood pressure, China, glucose metabolism, hypertension, insulin sensitivity, NAFLD

## Abstract

**Objective:**

This study aimed to describe the clinical characteristics of children and adolescents with obesity, and the prevalence of cardiometabolic comorbidities over 10 years in this population from a large metropolitan centre in China.

**Methods:**

This was a cross-sectional study (2008–2017) of patients aged <18 years with obesity [body mass index (BMI) ≥ 95th percentile for age and sex] enrolled at the Department of Endocrinology, Children’s Hospital of Zhejiang University School of Medicine (Hangzhou, Zhejiang Province). Clinical assessments included anthropometry, blood pressure, liver ultrasound, lipid profile, oral glucose tolerance test, and uric acid. For examination of outcomes, our study cohort was stratified by sex and age bands (<10 vs. ≥10 years), with the study period also split into two strata (2008–2012 and 2013–2017).

**Results:**

A total of 2,916 patients (1,954 boys and 962 girls) were assessed at a mean age of 10.5 years. Patients almost invariably presented severe obesity (median BMI SDS = 2.98; Q1 = 2.60, Q3 = 3.39). Obesity-related comorbidities were common among boys and girls, including type 2 diabetes mellitus (2.6% and 3.6%, respectively), abnormal glycaemia (33.6% and 35.5%, respectively), hypertension (33.9% and 32.0%, respectively), dyslipidaemia (35.2% and 39.6%, respectively), hyperuricaemia (16.2% and 8.3%, respectively), acanthosis nigricans (71.9% and 64.0%, respectively), abnormal liver function (66.9% and 47.0%, respectively), and non-alcoholic fatty liver disease (NAFLD) (63.8% and 45.1%, respectively); 38.7% of boys and 44.4% of girls aged ≥10 years had metabolic syndrome. Notably, the incidence of many cardiometabolic comorbidities was in 2013–2017 compared to 2008–2012. For example, rates of hypertension among boys aged <10 years and aged ≥10 years rose from 28.4% and 26.5% to 48.0% and 35.8%, respectively, and in girls from 20.3% and 20.8% to 41.7% and 39.6%, respectively. In 2013–2017, 9.5% of girls in the older group had metabolic syndrome compared to 2.2% in 2008–2013.

**Conclusions:**

We observed a high incidence of obesity-related cardiometabolic comorbidities among Chinese children and adolescents with severe obesity over 10 years. It was particularly concerning that rates of several comorbidities rose markedly over the study period, highlighting the need to address the obesity epidemic early in life (in China and elsewhere) to prevent the development of obesity-related comorbidities and, subsequently, of overt disease.

## Introduction

Worldwide, obesity is a major public health issue (1). The number of children and adolescents aged 5 to 19 years with obesity has risen 10-fold over the last four decades, reaching 124 million in 2016, with the global prevalence increasing from 0.7% to 5.6% in girls and from 0.9% to 7.8% in boys between 1975 and 2016, respectively (2). Notably, the mean body mass index (BMI) among children and adolescents has increased steadily, including in China (3). In 2015–2019, the prevalence of overweight and obesity in China was 6.8% and 3.6%, respectively, for children aged less than 6 years, and 11.1% and 7.9%, respectively, for those aged 6–17 years (4), with higher obesity rates reported in urban areas (5).

Children with obesity are at increased risk for cardiometabolic comorbidities, including hypertension, dyslipidaemia, hyperglycaemia, non-alcoholic fatty liver disease (NAFLD), and metabolic syndrome, which often track into adulthood with an increased risk of cardiovascular morbidity and mortality (6, 7). Of note, several paediatric definitions of metabolic syndrome agree on its components but differ in diagnostic criteria, and the International Diabetes Federation (IDF) definition seems to be more easily adopted in clinical practice (8). Moreover, the hazard ratio for type 2 diabetes mellitus is markedly elevated among adolescents with severe obesity (9). China is currently experiencing an accelerating diabetes epidemic as a result of a combination of factors (many of which interact), including increasing rates of obesity, changes in dietary habits (e.g., high in fat) and lifestyle (i.e., sedentary), ageing, and genetic and epigenetic factors (10). Of interest, a higher risk of diabetes at a lower BMI has been observed in the Chinese population compared to Europeans, likely resulting from the former’s greater visceral adiposity (11) and lower insulin response (12). Additionally, childhood obesity may favour early pubertal development or skeletal maturation and adverse psychosocial outcomes, including depression, anxiety, and eating disorder (13–15).

Therefore, childhood obesity and related cardiometabolic comorbidities are important issues that must be addressed. However, the incidence and severity of cardiometabolic comorbidities among Chinese children and adolescents with obesity have been overlooked in China over the last decades. Thus, our primary aim was to describe the clinical features of children and adolescents with obesity, particularly obesity-related cardiometabolic comorbidities. In addition, we also examined possible changes in the incidence of these comorbidities in boys and girls over the 10-year study period.

## Methods

### Study Participants

This was a cross-sectional study of children and adolescents voluntarily brought to our hospital by their parents who were concerned about excessive weight gain. These patients were then referred to the Department of Endocrinology at the Children’s Hospital of Zhejiang University School of Medicine, National Clinical Research Center for Child Health in Hangzhou, between January 1, 2008, and December 31, 2017. Hangzhou is the capital of Zhejiang Province, with a population of 6.77 million in 2008 and 7.53 million in 2017, including 1.05 million and 1.25 million children and adolescents aged ≤17 years, respectively (16, 17). The Children’s Hospital is one of only two National Clinical Research Centers for Child Health in China, recording approximately 81,000 inpatient and 3.5 million outpatient visits per year. Patients were only included once in this study, corresponding to their first visit to our clinic during the 10-year study period.

The main inclusion criterion was obesity at presentation, defined as a BMI SD score [SDS; derived as per the WHO standards (18) for age and sex] ≥1.645 (i.e., ≥95th percentile). At admission, none of our patients was on therapy with medications known to affect energy metabolism; had an overt chronic heart, lung, or kidney disease; or had been previously with diagnosed endocrine or metabolic dysfunction (e.g., Wilson’s disease), genetic disorders (e.g., Prader–Willi syndrome), or any severe chronic illness.

### Clinical Assessments

Participants were admitted to our inpatient clinic and underwent comprehensive clinical assessments over 24 h performed by nurses. Demographic characteristics were recorded or obtained from clinical records.

### Anthropometry

Standing height was measured to the nearest 0.1 cm using a wall-mounted Harpenden stadiometer while patients were barefoot. Weight was measured with the participant in light clothing using a digital scale to the nearest 0.1 kg; BMI was subsequently derived. Height, weight, and BMI were transformed into SDS (18). Waist circumference was measured to the nearest 1 mm with a tape measure around the participant’s body in the horizontal plane, at the midpoint level between the lowest rib and the iliac crest, on bare skin, and at the end of normal expiration. The waist-to-height ratio was then calculated. Maternal and paternal anthropometry data (i.e., height and weight) were obtained by self-report, and their BMI was calculated.

### Cardiometabolic Parameters

Systolic (SBP) and diastolic blood pressures (DBP) were measured using a sphygmomanometer on the right upper arm while patients seated and after a 5-min rest. Venous blood samples were taken on the morning of the assessment after an overnight fast. Parameters measured included glucose, insulin, glycated haemoglobin (HbA1c), triglycerides, low-density lipoprotein cholesterol (LDL), high-density lipoprotein cholesterol (HDL), total cholesterol, aspartate transaminase (AST), alanine transaminase (ALT), and uric acid.

All participants underwent a 75-g oral glucose tolerance test (OGTT; 1.75 g per kg, maximum 75 g), with blood samples drawn at 0, 30, 60, 90, and 120 min for glucose, insulin, and C-peptide measurements. Insulin sensitivity was assessed using the Matsuda index, which is strongly correlated with the hyperinsulinaemic–euglycaemic clamp and has high reproducibility during multiple measures (19).

Cardiometabolic comorbidities assessed included the following: impaired fasting glucose, impaired glucose tolerance, type 2 diabetes, and abnormal glycaemia (20); hypertension (21); dyslipidaemia (22); metabolic syndrome (22); NAFLD (23); and hyperuricaemia (24) (**Table 1**). Acanthosis nigricans was also recorded, as it is a clinical sign of hyperinsulinaemia and insulin resistance (26, 27). Central obesity was defined as per Chinese criteria for children and adolescents based on the waist-to-height ratio (21) (**Table 1**).

**Table 1:** Diagnostic criteria for central adiposity and obesity-related cardiometabolic comorbidities assessed.

Condition	Age	Minimum diagnostic criteria	Reference
**Impaired fasting glucose**	All	Fasting plasma glucose ≥5.6 and <7.0 mmol/L	Arslanian et al. (20)
**Impaired glucose tolerance**	All	a) Fasting plasma glucose <7.0 mmol/L; AND b) 2-h plasma glucose ≥7.8 and <11.1 mmol/L^#^	Arslanian et al. (20)
**Type 2 diabetes**	All	a) Fasting plasma glucose ≥7.0 mmol/L; OR b) 2-h plasma glucose ≥11.1 mmol/L^#^	Arslanian et al. (20)
**Abnormal glycaemia**	All	a) Impaired fasting glucose; OR b) Impaired glucose tolerance; OR c) Type 2 diabetes	Arslanian et al. (20)
**Hypertension**	<10 years	a) Systolic blood pressure ≥120 mmHg; OR b) Diastolic blood pressure ≥80 mmHg	Chinese Medical Association (21)
	≥10 years	a) Systolic blood pressure ≥130 mmHg; OR b) Diastolic blood pressure ≥85 mmHg	Chinese Medical Association (21)
**Dyslipidaemia**	All	a) Triglycerides ≥1.7 mmol/L; OR b) HDL <1.03 mmol/L	Zimmet et al. (22)
**Metabolic syndrome**	≥10 years	a) Obesity; AND b) any two of: • Abnormal glycaemia (as defined above; • Hypertension (as defined above); • Dyslipidaemia (as defined above);	Zimmet et al. (22)
**Hyperuricaemia**	All	Plasma uric acid ≥5.5 mg/dl	Loeffler et al. (24)
**NAFLD**	All	“A diffusely echogenic change in liver B-ultrasonography, with or without elevated serum aminotransferase levels and other factors that can cause liver fatty infiltration or aminotransferase elevation, such as hepatitis virus infection, drug-induced injury, and other metabolic diseases, such as Wilson’s disease, were excluded.”	Chinese Liver Disease Assoc (23).
**Central obesity**	≥6 and <10 years	Waist-to-height ratio ≥0.48	Chinese Medical Association (21)
	≥10 and <16 years	Boys: Waist-to-height ratio ≥0.48 Girls: Waist-to-height ratio ≥0.46	Chinese Medical Association (21)

Adapted from Jin et al. (25).

HDL, high-density lipoprotein cholesterol; NAFLD, non-alcoholic fatty liver disease.

^#^Parameter measured after a 75-g glucose load from an oral glucose tolerance test.

### Statistical Analyses

Descriptive data for demographic and anthropometric characteristics of our overall study population are provided as means ± SDs or frequency (n) and percentages (%). The incidence of central obesity and obesity-related cardiometabolic comorbidities is provided as n (%).

Patients were then stratified according to the year of admission (into two 5-year study periods: 2008–2012 and 2013–2017), sex (male and female), and age (<10 and ≥10 years). Within each age group, the incidence of comorbidities was compared between sexes and between study periods using Fisher’s exact tests.

Continuous outcomes for anthropometry, blood pressure, glucose metabolism, and lipid profile were also compared between the groups mentioned above. Potential differences were assessed using general linear regression models adjusting for the participant’s age, with the latter replaced with height for blood pressure outcomes. The data distribution of each outcome was examined, and, where appropriate, data were log-transformed to approximate a normal distribution. For continuous variables, differences between sexes or study periods are reported in the text as the estimated marginal means (adjusted means) and 95% CIs.

Analyses were performed in SPSS v25 (IBM Corp., Armonk, NY, USA) and SAS v9.4 (SAS Institute, Cary, NC, USA). All tests were two-tailed, with statistical significance maintained at p < 0.05, and without adjustment for multiple comparisons as per Rothman (1990) (28).

## Results

**Figure 1:**
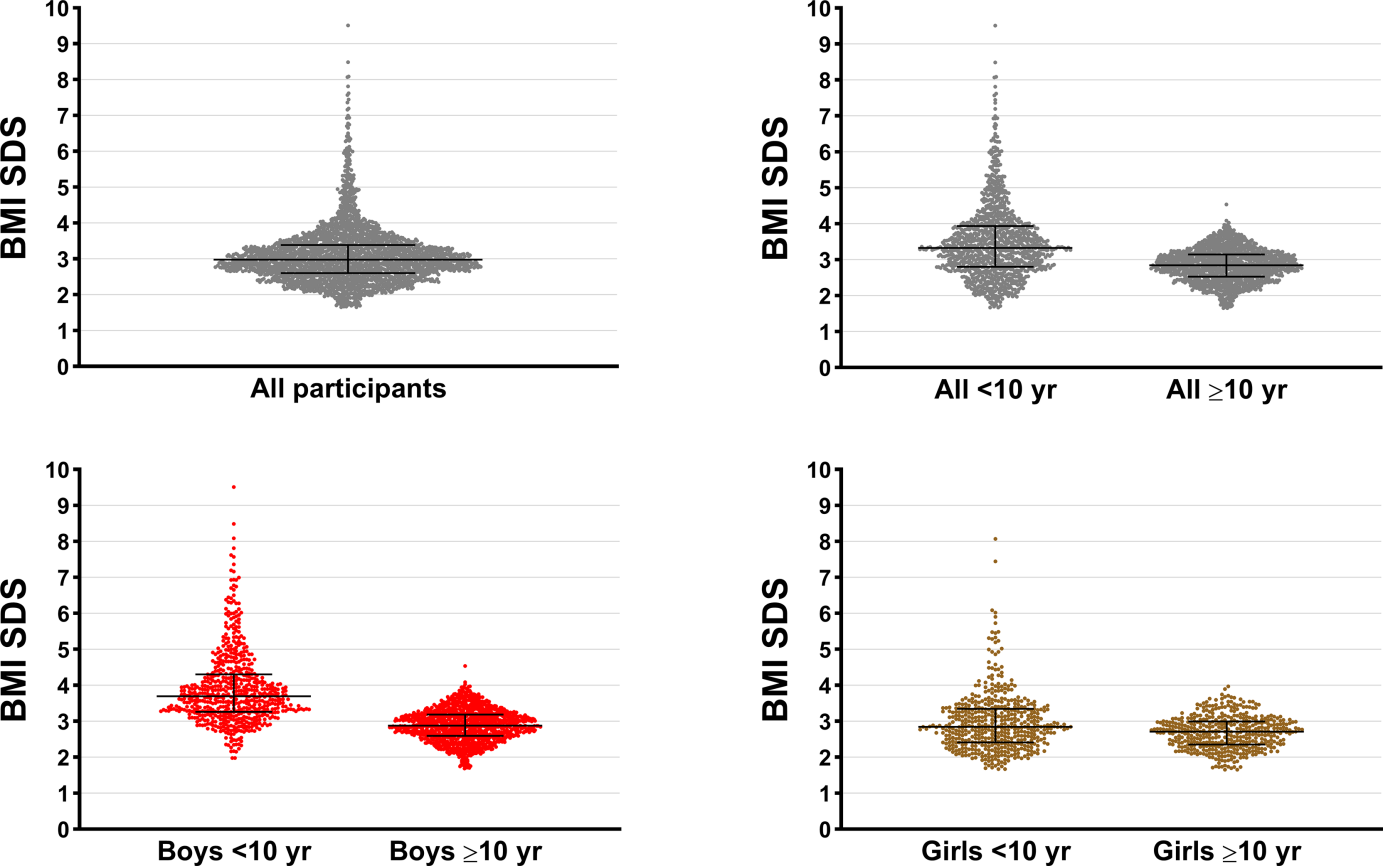
Distribution of body mass index SD scores (BMI SDS) among our study population of children and adolescents with obesity assessed in 2008–2017 in Hangzhou (Zhejiang Providence, China). Horizontal bars represent the median and the interquartile range.

Our study population consisted of 2,916 children and adolescents with obesity, assessed at a mean age of 10.5 ± 2.6 years, including 1,954 boys and 962 girls (**Table 2**). Participants had a median BMI SDS of 2.98 (Q1 = 2.60, Q3 = 3.39; range 1.65–9.51), and the vast majority had severe obesity (**Figure 1**). Among caregivers, 1 in 3 mothers (33.2%) and more than half of fathers (54.7%) had overweight or obesity (**Table 2**).

**Table 2:** Demographic, anthropometric, and clinical characteristics of our study population of children and adolescents with obesity from Hangzhou (Zhejiang Province, China) in 2008–2017.

n		2,916
**Demography**	**Age (years)**	10.5 ± 2.6
	**Female**	962 (33.0%)
**Anthropometry**	**Height SDS**	1.02 ± 1.18
	**Weight SDS**	3.39 ± 1.02
	**BMI SDS**	3.08 ± 0.82
	**Waist-to-height ratio**	0.604 ± 0.057
**Parental characteristics ^1^**	**Maternal BMI (kg/m^2^)**	24.00 ± 3.52
	**Maternal obesity**	166 (5.9%)
	**Maternal overweight or obesity**	969 (33.2%)
	**Paternal BMI (kg/m^2^)**	25.76 ± 3.50
	**Paternal obesity**	295 (10.6%)
	**Paternal overweight or obesity**	1,596 (54.7%)

Data are the mean ± SD or n (%), as appropriate.

BMI, body mass index; SDS, SD scores.

^1^The total n for maternal and paternal BMI was 2,799 and 2,793, respectively.

As shown in **Table 3**, there was a high incidence of cardiometabolic comorbidities among children and adolescents with obesity in China over the 10-year period covered by this study. Among boys and girls, these included type 2 diabetes (2.6% and 3.6%, respectively), abnormal glycaemia (33.6% and 35.5%), hypertension (33.9% and 32.0%), and dyslipidaemia (35.2% and 39.6%), with almost all boys (99.6%) and girls (98.5%) having central obesity (**Table 3**).

**Table 3:** Incidence of cardiometabolic comorbidities among children and adolescents with obesity assessed between 2008 and 2017 in Hangzhou (Zhejiang Province, China).

	All ages			Aged <10 years			Aged ≥10 years		
	All	Boys	Girls	All	Boys	Girls	All	Boys	Girls
**N**	2,916	1,954	962	1,132	623	509	1,784	1,331	453
**IFG**	722 (24.8%)	481 (24.7%)	241 (25.1%)	275 (24.4%)	154 (24.9%)	121 (23.8%)	447 (25.1%)	327 (24.6%)	120 (26.5%)
**IGT**	432 (14.9%)	282 (14.5%)	150 (15.6%)	137 (12.2%)	82 (13.2%)	55 (10.8%)	295 (16.6%)	200 (15.1%)	95 (21.0%)**
**Abnormal glycaemia**	996 (34.3%)	655 (33.6%)	341 (35.5%)	361 (32.0%)	205 (33.1%)	156 (30.7%)	635 (35.7%)	450 (33.9%)	185 (40.8%)**
**Type 2 diabetes**	85 (2.9%)	50 (2.6%)	35 (3.6%)	10 (0.9%)	5 (0.8%)	5 (1.0%)	75 (4.2%)	45 (3.4%)	30 (6.6%)**
**Hypertension**	968 (33.3%)	660 (33.9%)	308 (32.0%)	402 (35.6%)	240 (38.6%)	162 (31.9%)*	566 (31.8%)	420 (31.6%)	147 (32.2%)
**Dyslipidaemia**	1,058 (36.7%)	684 (35.2%)	374 (39.6%)*	348 (31.1%)	184 (29.7%)	164 (32.7%)	710 (40.2%)	500 (37.8%)	210 (47.3%)***
**Hyperuricaemia**	391 (13.7%)	313 (16.2%)	78 (8.3%)****	56 (5.1%)	32 (5.2%)	24 (4.9%)	335 (19.1%)	281 (21.4%)	54 (12.2%)****
**Acanthosis nigricans**	1,980 (69.3%)	1,378 (71.9%)	602 (64.0%)****	673 (60.9%)	412 (67.5%)	261 (52.7%)****	1,307 (74.6%)	966 (73.9%)	341 (76.5%)
**Abnormal liver function**	1,733 (60.4%)	1,292 (66.9%)	441 (47.0%)****	551 (49.7%)	358 (58.3%)	193 (39.1%)****	1,182 (67.1%)	934 (70.9%)	248 (55.9%)****
**NAFLD**	1,678 (57.6%)	1,245 (63.8%)	433 (45.1%)****	477 (42.2%)	313 (50.3%)	164 (32.3%)****	1,201 (67.4%)	932 (70.2%)	269 (59.4%)****
**Metabolic syndrome**	–	–	–	–	–	–	716 (40.1%)	515 (38.7%)	201 (44.4%)*
**Central obesity^1^**	2,736 (99.2%)	1,853 (99.6%)	883 (98.5%)	962 (98.9%)	528 (99.6%)	434 (98.0%)	1,774 (99.4%)	1,325 (99.5%)	449 (99.1%)

IFG, Impaired fasting glucose; IGT, Impaired glucose tolerance; NAFLD, non-alcoholic fatty liver disease.

^1^Central obesity as per Chinese standards is not diagnosed in children aged less than 6 years (21); therefore, 93 boys and 56 girls were excluded from the calculation of these rates.

Data are n (%).

*p < 0.05, **p < 0.01, ***p < 0.001, and ****p < 0.0001 for comparisons in incidence of a given comorbidity within a particular group.

There was a greater proportion of boys than girls with hyperuricaemia (16.2% vs. 8.3%, respectively; p < 0.0001), acanthosis nigricans (71.9% vs. 64.0%; p < 0.0001), abnormal liver function (66.9% vs. 47.0%; p < 0.0001), and NAFLD (63.8% vs. 45.1%; p < 0.0001), with these sex differences largely observed in the two age groups (**Table 3**). Among children aged <10 years, there was a greater incidence of hypertension in boys than girls (38.6% vs. 31.9%; p = 0.021) (**Table 3**). In addition, the incidence of obesity-related comorbidities was greater among patients aged ≥10 years than in the younger group (**Table 3**). Notably, 38.7% of boys and 44.4% of girls aged ≥10 years had metabolic syndrome (**Table 3**).

When the two 5-year periods (2008–2012 vs. 2013–2017) were compared, among the younger boys, there was a higher incidence of impaired fasting glucose, abnormal glycaemia, acanthosis nigricans, and, in particular, hypertension (**Table 4**). These differences were underpinned by higher SBP (+6.2 mmHg; 95% CI 4.3, 8.0 mmHg), DBP (+2.3 mmHg; 95% CI 1.0, 3.7 mmHg), fasting glucose (+0.20 mmol/L; 95% CI 0.13, 0.27 mmol/L), and a Matsuda index that was 18% lower (95% CI −1.3%, −31.6%) in 2013–2017 (**Table 5**). Conversely, the incidence of dyslipidaemia was lower in the later period, likely associated with HDL +7.3% higher (+0.09 mmol/L; 95% CI 0.05, 0.14 mmol/L) and possibly slightly lower triglycerides (**Table 5**).

**Table 4:** Incidence of cardiometabolic comorbidities among Chinese boys with obesity assessed between 2008 and 2017 in Hangzhou (Zhejiang Province, China).

Boys	Aged <10 years			Aged ≥10 years		
	2008–2012	2013–2017	p-Value	2008–2012	2013–2017	p-Value
**N**	298	325		600	731	
**Impaired fasting glucose**	59 (20.1%)	95 (29.2%)	**0.009**	132 (22.1%)	195 (26.7%)	0.055
**Impaired glucose tolerance**	38 (12.9%)	44 (13.5%)	0.91	76 (12.8%)	124 (17.0%)	**0.037**
**Abnormal glycaemia**	78 (26.5%)	127 (39.1%)	**0.001**	174 (29.1%)	276 (37.8%)	**0.001**
**Type 2 diabetes**	1 (0.3%)	4 (1.2%)	0.38	9 (1.5%)	36 (4.9%)	**0.001**
**Hypertension**	84 (28.4%)	156 (48.0%)	**<0.0001**	158 (26.5%)	262 (35.8%)	**<0.0001**
**Dyslipidaemia**	105 (35.4%)	79 (24.5%)	**0.004**	253 (42.4%)	247 (34.2%)	**0.002**
**Hyperuricaemia**	14 (4.7%)	18 (5.6%)	0.72	125 (21.2%)	156 (21.5%)	0.95
**Acanthosis nigricans**	176 (61.5%)	236 (72.8%)	**0.003**	414 (69.3%)	518 (70.9%)	0.59
**Abnormal liver function**	191 (65.0%)	167 (52.2%)	**0.001**	393 (68.0%)	573 (78.6%)	**<0.0001**
**NAFLD**	155 (52.2%)	158 (48.6%)	0.38	434 (73.4%)	500 (68.8%)	0.07
**Metabolic syndrome**	–	–	–	222 (37.0%)	293 (40.1%)	0.26

Data are n (%). p-Values are derived from Fisher’s exact tests, and correspond to comparisons in the incidence of a particular comorbidity between time periods within a given age group. Statistically significant p-values (<0.05) are shown in bold.

NAFLD, non-alcoholic fatty liver disease.

**Table 5:** Cardiometabolic parameters among Chinese boys with obesity assessed between 2008 and 2017 in Hangzhou (Zhejiang Province, China).

Boys		Aged <10 years			Aged ≥10 years		
		2008–2012	2013–2017	p-Value	2008–2012	2013–2017	p-Value
**n**		298	325		600	731	
**Age**		7.9 ± 1.7	8.0 ± 1.7	0.36	11.9 ± 1.4	12.1 ± 1.4	0.15
**Anthropometry**	**Height SDS**	1.38 ± 1.16	1.44 ± 1.13	0.51	0.85 ± 1.19	0.95 ± 1.17	**0.031**
	**BMI SDS**	3.90 ± 1.05	3.92 ± 1.02	0.17	2.88 ± 0.44	2.90 ± 0.44	0.17
	**Waist-to-height ratio**	0.624 ± 0.059	0.627 ± 0.059	0.33	0.604 ± 0.048	0.609 ± 0.053	**0.042**
**Blood pressure**	**Systolic (mmHg)**	112.4 ± 12.7	118.8 ± 11.9	**<0.0001**	119.6 ± 14.7	124.5 ± 13.3	**<0.0001**
	**Diastolic (mmHg)**	67.4 ± 8.2	69.9 ± 9.1	**<0.001**	69.3 ± 8.6	71.7 ± 10.0	**<0.0001**
**Glucose metabolism**	**Matsuda index**	75.9 (62.2, 92.8)	60.9 (56.3, 66.0)	**0.036**	49.4 (43.8, 55.7)	41.7 (39.6, 43.4)	**0.005**
	**HbA1c (%)**	5.85 ± 0.57	5.76 ± 0.71	0.08	5.85 ± 0.54	5.82 ± 0.63	0.43
	**Fasting glucose (mmol/L)**	5.21 ± 0.53	5.41 ± 0.38	**<0.0001**	5.25 ± 0.61	5.38 ± 0.45	**<0.0001**
	**Fasting insulin (μIU/ml)**	10.7 (9.7, 11.9)	12.0 (10.9, 13.2)	0.11	15.8 (14.9, 16.9)	18.1 (17.1, 19.2)	**0.002**
**Lipid profile**	**Total cholesterol (mmol/L)**	4.28 ± 0.73	4.33 ± 0.81	0.47	4.41 ± 0.82	4.34 ± 0.90	0.16
	**Triglycerides (mmol/L)**	1.15 (1.10, 1.21)	1.08 (1.03, 1.14)	0.08	1.25 (1.21, 1.30)	1.26 (1.22, 1.30)	0.87
	**LDL (mmol/L)**	2.32 ± 0.54	2.68 ± 0.61	**<0.0001**	2.51 ± 0.66	2.74 ± 0.68	**<0.0001**
	**HDL (mmol/L)**	1.23 ± 0.28	1.32 ± 0.26	**<0.0001**	1.23 ± 0.31	1.25 ± 0.26	0.11
	**Total cholesterol/HDL**	3.62 ± 0.94	3.36 ± 0.80	**<0.0001**	3.80 ± 1.17	3.59 ± 1.02	**<0.0001**
**Inflammatory marker**	**Uric acid (μmol/ml)**	3.89 ± 0.93	3.97 ± 0.86	0.31	4.57 ± 1.13	4.68 ± 1.12	0.17

For Matsuda index, fasting insulin, and triglycerides the back-transformed data are reported as means and respective 95% CIs; all other data are means ± SDs. Statistically significant p-values (<0.05) are shown in bold.

BMI, body mass index; HbA1c, glycated haemoglobin; HDL, high-density lipoprotein cholesterol; LDL, low-density lipoprotein cholesterol; SDS, SD score.

In the older group of boys, in 2013–2017, there was a higher incidence of hypertension, abnormal liver function, and abnormal glycaemia, including type 2 diabetes (4.9% vs. 1.5%; **Table 4**). As seen among the younger boys, these comorbidities were more frequent in the later period in association with higher SBP (+4.3 mmHg; 95% CI 2.9, 5.8 mmHg), DBP (+2.3 mmHg; 95% CI 1.3, 3.4 mmHg), fasting glucose (+0.13 mmol/L; 95% CI 0.07, 0.19 mmol/L), fasting insulin (+14%; 95% CI 5%, 24%), and lower Matsuda index (−15%; 95% CI −5%, −25%) in 2013–2017 (**Table 5**).

Among the younger girls, there were proportionally fewer patients with abnormal liver function in 2013–2017 (**Table 6**), but the rate of hypertension was 2-fold higher (41.7% vs. 20.3%), which was associated with higher SBP (+9.2 mmHg; 95% CI 7.2, 11.3 mmHg) and DBP (+2.6 mmHg; 95% CI 1.0, 4.2 mmHg) (**Table 7**). In addition, girls in the second period had fasting glucose concentrations 0.15 mmol/L higher (95% CI 0.04, 0.26 mmol/L) and Matsuda index 28% lower (95% CI −10%, −42%) (**Table 7**).

**Table 6:** Incidence of cardiometabolic comorbidities among Chinese girls with obesity assessed between 2008 and 2017 in Hangzhou (Zhejiang Province, China).

Girls	Aged <10 years			Aged ≥10 years		
	2008–2012	2013–2017	p-Value	2008–2012	2013–2017	p-Value
**N**	232	276		178	275	
**Impaired fasting glucose**	56 (24.1%)	65 (23.6%)	0.92	42 (23.6%)	78 (28.4%)	0.28
**Impaired glucose tolerance**	24 (10.3%)	31 (11.2%)	0.78	39 (21.9%)	56 (20.4%)	0.72
**Abnormal glycaemia**	73 (31.5%)	83 (30.1%)	0.77	66 (37.1%)	119 (43.3%)	0.20
**Type 2 diabetes**	nil	5 (1.8%)	0.07	4 (2.2%)	26 (9.5%)	**0.002**
**Hypertension**	47 (20.3%)	115 (41.7%)	**<0.0001**	37 (20.8%)	109 (39.6%)	**<0.0001**
**Dyslipidaemia**	84 (36.5%)	80 (29.5%)	0.10	93 (52.5%)	117 (43.8%)	0.08
**Hyperuricaemia**	12 (5.3%)	12 (4.5%)	0.68	15 (8.7%)	39 (14.5%)	0.08
**Acanthosis nigricans**	106 (48.0%)	155 (56.6%)	0.058	118 (68.2%)	223 (81.7%)	**0.001**
**Abnormal liver function**	104 (46.0%)	89 (33.2%)	**0.004**	88 (50.6%)	160 (59.3%)	0.08
**NAFLD**	84 (36.2%)	80 (29.0%)	0.09	103 (57.9%)	166 (60.4%)	0.63
**Metabolic syndrome**	–	–	–	74 (41.6%)	127 (46.2%)	0.38

Data are n (%). p-Values are derived from chi-square tests or Fisher’s exact tests; they correspond to comparisons in the incidence of particular comorbidity between time periods within a given age group. Statistically significant p-values (<0.05) are shown in bold.

NAFLD, non-alcoholic fatty liver disease.

**Table 7:** Cardiometabolic parameters among girls with obesity assessed between 2008 and 2017 in Hangzhou (Zhejiang Province, China).

Girls		Aged <10 years			Aged ≥10 years		
		2008–2012	2013–2017	p-Value	2008–2012	2013–2017	p-Value
**n**		232	276		178	275	
**Age**		7.9 ± 1.4	7.9 ± 1.3	0.88	12.3 ± 1.7	12.6 ± 1.7	0.13
**Anthropometry**	**Height SDS**	1.26 ± 1.08	1.35 ± 1.02	0.36	0.50 ± 1.08	0.60 ± 1.15	0.15
	**BMI SDS**	3.04 ± 0.87	2.91 ± 0.74	**0.015**	2.67 ± 0.43	2.71 ± 0.47	0.68
	**Waist-to-height ratio**	0.587 ± 0.061	0.577 ± 0.052	**0.026**	0.587 ± 0.057	0.601 ± 0.058	**0.046**
**Blood pressure**	**Systolic (mmHg)**	108.0 ± 12.4	117.4 ± 12.1	**<0.0001**	118.5 ± 14.1	124.0 ± 12.0	**<0.0001**
	**Diastolic (mmHg)**	65.5 ± 8.1	68.2 ± 9.9	**0.002**	68.3 ± 8.5	73.1 ± 9.9	**<0.0001**
**Glucose metabolism**	**Matsuda index**	90.0 (71.5, 114.4)	63.4 (58.0, 69.4)	**0.004**	56.3 (41.7, 76.7)	32.5 (30.3, 35.2)	**<0.0001**
	**HbA1c (%)**	5.74 ± 0.57	5.66 ± 0.55	0.07	5.87 ± 0.56	5.78 ± 0.71	0.21
	**Fasting glucose (mmol/L)**	5.24 ± 0.57	5.39 ± 0.67	**0.007**	5.27 ± 0.78	5.38 ± 0.71	0.07
	**Fasting insulin (μIU/ml)**	11.6 (10.3, 13.1)	12.1 (10.9, 13.5)	0.62	18.1 (16.0, 20.5)	23.2 (21.0, 25.6)	**0.003**
**Lipid profile**	**Total cholesterol (mmol/L)**	4.29 ± 0.92	4.11 ± 0.84	**0.033**	4.25 ± 0.88	4.30 ± 0.94	0.53
	**Triglycerides (mmol/L)**	1.51 ± 1.67	1.28 ± 0.73	0.07	1.47 ± 0.76	1.52 ± 0.77	0.45
	**LDL (mmol/L)**	2.43 ± 0.71	2.55 ± 0.58	**0.028**	2.48 ± 0.70	2.74 ± 0.67	**<0.0001**
	**HDL (mmol/L)**	1.22 ± 0.31	1.28 ± 0.28	**<0.0001**	1.11 ± 0.25	1.17 ± 0.24	**0.004**
	**Total cholesterol/HDL**	3.74 ± 1.27	3.35 ± 0.91	**<0.0001**	3.97 ± 1.08	3.77 ± 0.97	**0.033**
**Inflammatory marker**	**Uric acid (μmol/ml)**	3.88 ± 0.89	3.92 ± 0.90	0.65	4.42 ± 0.84	4.54 ± 1.01	0.34

For Matsuda index, fasting insulin, and triglycerides the back-transformed data are reported as means and respective 95% CIs; all other data are means ± SDs. Statistically significant p-values (<0.05) are shown in bold.

BMI, body mass index; HbA1c, glycated haemoglobin; HDL, high-density lipoprotein cholesterol; LDL, low-density lipoprotein cholesterol; SDS, SD score.

For girls aged 10 years or older, there was a higher incidence of acanthosis nigricans, hypertension, and a 4.5-fold higher incidence of type 2 diabetes affecting nearly 10% of girls in 2013–2017 (9.5% vs. 2.2%) (**Table 6**). In this older group of girls, there were other clinical parameters worse in 2013–2017 than in 2008–2012, including higher waist-to-height ratio (+0.011; 95% CI 0.000, 0.022), SBP (+4.8 mmHg; 95% CI 2.4, 7.2 mmHg), DBP (+4.6 mmHg; 95% CI 2.8, 6.3 mmHg), LDL (+0.26 mmol/L; 95% CI 0.13, 0.39 mmol/L), and fasting insulin (+28%; 95% CI 9%, 50%) but lower Matsuda index (−42%; 95% CI −26%, −54%) (**Table 7**). Conversely, HDL was higher (+0.07 mmol/L; 95% CI 0.02, 0.11 mmol/L) and the cholesterol:HDL ratio lower (−0.21; 95% CI −0.41, −0.02) (**Table 7**).

## Discussion

To our knowledge, this was the largest single-centre cross-sectional study (n = 2,916) to comprehensively assess obesity-related comorbidities among children and adolescents with severe obesity in China. There was a high incidence of a range of cardiometabolic comorbidities, including abnormal glycaemia, hypertension, dyslipidaemia, hyperuricaemia, acanthosis nigricans, abnormal liver function, and NAFLD. These findings are not surprising given the strong association between higher BMI during adolescence and increased risk for cardiometabolic complications (15, 29). Our paediatric population characterised by very high BMI SDS illustrates the extent of the potential impacts of childhood obesity (and related comorbidities) on public health in China and elsewhere, a growing challenge that has been previously underestimated (30, 31).

In 2013, the prevalence of metabolic syndrome in Chinese children with obesity was 28.8% (32), in contrast to our observed rates of 40.1% and 46.2% for boys and girls, respectively, in 2013–2017. The much higher incidence in our study population is not surprising given that most of our patients had severe obesity. Other common comorbidities in our study included abnormal liver function, NAFLD, and acanthosis nigricans. The latter, in particular, is highly prevalent and a specific clinical sign of insulin resistance, a key component of the metabolic syndrome (33, 34). Our patients also had insulin sensitivity assessed with the Matsuda index, a robust measure to examine abnormalities in glucose metabolism in children and adolescents with obesity (35). Previous studies have also shown high rates of disorders in glucose metabolism among asymptomatic children and adolescents with obesity (36). Here, we also observed an apparent reduction in insulin sensitivity and an associated increase in fasting glucose levels between the two 5-year periods (2008–2012 vs. 2013–2017); these findings suggest that children with severe obesity are displaying increasing levels of impairment in glucose metabolism and possibly increased risk of progressing to type 2 diabetes.

The growing problem of childhood obesity seems to be underpinning the increasing burden of NAFLD. In our study, 2 out of 3 boys aged 10 years or older displayed liver function abnormalities and/or were affected by NAFLD. In a study on ≈15,000 children and adolescents from 6 centres throughout China, we identified elevated liver enzymes associated with metabolic syndrome features, highlighting the role of chronic insulin resistance and metabolic syndrome in the aetiology of liver injury in Chinese youth (37). While we have no data on the clinical history of our patients (particularly in regard to weight gain over time), our findings are not surprising given that, after 2 years of age, obesity progressively increases the risk of developing NAFLD in adolescence (38). Therefore, it is important to monitor liver function and the potential development of NAFLD over time among children and adolescents with obesity, specifically liver function tests and liver ultrasound (39, 40).

The link between obesity and hypertension is well established (41, 42). The reported prevalence of hypertension in schoolchildren with obesity was approximately 11% in the United States (43). In Greece, a study on 2,655 schoolchildren aged 9–13 years showed higher rates of hypertension of 25.3% and 20.8% for girls and boys, respectively (44). Among Chinese youth, Cao (2009) reported rates of hypertension of 11.5% in girls and 21.7% in boys with obesity aged 12 to 17 years (45). The higher rates of hypertension among our patients (33.3%) would be expected given their obesity severity, as alluded to earlier. For example, Lo et al. showed that the odds of hypertension were 2.7 times greater in children with severe obesity compared to those with moderate obesity (46). Notably, the incidence of hypertension increased between 2008–2012 and 2013–2017 among our patients, irrespective of sex or age, reflecting corresponding increases in SBP and DBP over time. The reasons for the changing incidence of hypertension over time are unclear, particularly in the absence of differences in obesity levels. It is possible that changes in dietary habits and physical activity levels could explain, at least in part, the worsening rates of hypertension in the latest period. However, this cannot be ascertained in our study population, as such information was not recorded. Nonetheless, independent of the underlying causes of our observed trend, blood pressure should be monitored in the long-term (47) among paediatric patients with obesity, as a longer duration of hypertension increases the cardiovascular risk and end-organ damage (48).

Of note, the ratio of boys to girls in our study was approximately 2:1. It is plausible that this could reflect some bias among parents, who would be more likely to identify weight issues in boys than in girls. Previously, in a study of more than 20,000 children and adolescents from 6 centres across China, we reported that parents were more likely to overestimate the BMI status of girls compared to boys (49). However, the child’s sex was not associated with the parents’ ability to correctly identify an obesity issue or, most importantly in the context of the present study, of seeking treatment for their child if weight issues were identified (49). Most likely, the overrepresentation of boys in our study is simply a reflection of the greater prevalence of obesity in boys in the general population: in 2013, the prevalence of obesity in children and adolescents in China was 6.9% among boys and 2.8% among girls (50).

The main limitation of our study was the lack of complete data on pubertal development, which was only recorded on approximately 80% of our study population; thus, our study cohort was stratified using a relatively arbitrary age threshold, as used for the classification of the metabolic syndrome (22). While there was no selection bias by study investigators since all patients were self-reported (by their parents), the BMI SDS 10th percentile among our patients was 2.27, illustrating that our study population was skewed towards the upper end of the BMI SDS spectrum, consisting primarily of individuals with severe obesity. This is not surprising, as we have shown that Chinese caregivers are more likely to identify problems with excess weight in children with severe obesity (49). Nonetheless, this means that our observed incidence of obesity-related comorbidities cannot be readily extrapolated to children and adolescents with more moderate levels of obesity. Another limitation of our study was a potential regional bias; our patients were assessed in east China, and the prevalence of obesity-related comorbidities may differ in other regions of the country. Lastly, this was a cross-sectional study, as longitudinal data were not available; since comparisons between the two study periods were made between two different groups of patients, we cannot ascertain the progression of obesity-related comorbidities in individual patients over time. Nevertheless, our study is particularly valuable due to the comprehensive range of clinical assessments performed (e.g., blood pressure, OGTT, and liver ultrasound) and the large number of children and adolescents assessed.

In conclusion, our study shows a high incidence of obesity-related cardiometabolic comorbidities among Chinese children and adolescents with severe obesity over 10 years, including hypertension, NAFLD, and abnormalities in glucose metabolism. It was particularly concerning that rates of several comorbidities rose markedly over the study period, highlighting the need to address the obesity epidemic early in life in China and elsewhere to prevent the development of obesity-related comorbidities and, subsequently, of overt disease.

## Data Availability Statement

The data presented in this article are not readily available because of the conditions of the ethics approval. The anonymized data on which this article was based could be made available to other investigators upon bona fide request, and following all the necessary approvals (including ethics) of the detailed study proposal and statistical analyses plan. Requests to access the dataset should be directed to Prof. Junfen Fu, fjf68@zju.edu.cn.

## Ethics Statement

This study was approved by the Medical Ethics Committee of the Children's Hospital of Zhejiang University School of Medicine (No. 2020-IRB-098). Written informed consent was obtained from parents (or caregivers) and verbal or written consent from each child as appropriate to their age. This study was performed following all applicable institutional and international guidelines and regulations for medical research, in line with the principles of the Declaration of Helsinki (51).

## Author Contributions

JF was responsible for funding acquisition. JF, JD, JW, HL, BJ, and JY contributed to the study design. JW, HL, JY, KH, WW, and GD carried out the clinical assessments. JW, HL, JY, BJ, and JD were responsible for data curation and analyses, with results critically reviewed by JF, VC, KH, WW, and GD. JW, VC, and JD wrote the manuscript with critical input from all other authors. All authors listed have made a substantial, direct, and intellectual contribution to the work and approved it for publication.

## Funding

This work was supported by the National Key Research and Development Program of China (No. 2021YFC2701901 and No. 2016YFC1305301), the National Natural Science Foundation of China (No. 81570759 and 81270938), the Fundamental Research Funds for the Central Universities (2020XZZX002-22), the Research Fund of Zhejiang Major Medical and Health Science and Technology and National Ministry of Health (WKJ-ZJ-1804), Zhejiang Provincial Natural Science Foundation of China (LQ20H070003), Zhejiang Provincial Key Disciplines of Medicine (Innovation Discipline, 11-CX24), and Zhejiang Province Natural Sciences Foundation Zhejiang Society for Mathematical Medicine (LSZ19H070001). JD was supported by a travel fellowship from the New Zealand–China Non-Communicable Diseases Research Collaboration Centre.

## Conflict of Interest

The authors declare that the research was conducted in the absence of any commercial or financial relationships that could be construed as a potential conflict of interest.

## Publisher’s Note

All claims expressed in this article are solely those of the authors and do not necessarily represent those of their affiliated organizations, or those of the publisher, the editors and the reviewers. Any product that may be evaluated in this article, or claim that may be made by its manufacturer, is not guaranteed or endorsed by the publisher.
